# COVID-19 Worry and Mental Health Among the Economically Active Population in Guangdong, China

**DOI:** 10.3389/fpubh.2022.882177

**Published:** 2022-05-02

**Authors:** Xin Yong, Li Zhang

**Affiliations:** ^1^School of Public Administration, Xiangtan University, Xiangtan, China; ^2^School of Economics and Trade, Hunan University, Changsha, China

**Keywords:** COVID-19 pandemic, worry, mental health, economically active population, GHQ-12

## Abstract

**Background:**

The rapid spread of the coronavirus disease 2019 (COVID-19) pandemic has caused people to worry, which has affected their mental health. This study aimed to access the impact of COVID-19 worry on the mental health of the economically active population (EAP) in a province of China.

**Methods:**

An online cross-sectional survey study was conducted during an outbreak of COVID-19 in Guangdong, China. The survey used the 12-item General Health Questionnaire (GHQ-12) to evaluate participants' mental health status and was completed by 1,584 of the 1,708 participants (a response rate of 92.74%). Ordinary least squares (OLS) regression models were used to identify the correlation between COVID-19 worry and mental health.

**Results:**

Approximately 42.05% of participants reported that they were very worried or extremely worried about the COVID-19 pandemic. COVID-19 worry was negatively correlated with mental health (*p* < 0.01) and exhibited a stronger influence on the mental health of participants who were male, younger (aged 16–45), or unemployed than on the mental health of participants who were women, older (aged over 45), or employed.

**Conclusion:**

The findings suggest that COVID-19 worry has generated new inequalities in mental health among the EAP of China. The government should provide more public reassurance and psychological support to the EAP to mitigate the effects of COVID-19 worry and prevent mental health disorders.

## Introduction

Coronavirus disease 2019 (COVID-19), which is caused by a serious infection of severe acute respiratory syndrome coronavirus 2 (SARS-CoV-2), originated in Wuhan, China, at the end of December 2019, and rapidly spread to become a nationwide epidemic and thereafter a global pandemic. The Chinese government has adopted a “zero tolerance” policy in efforts to contain the pandemic, including the use of severe measures, such as abrupt lockdowns of cities and strict home quarantine. For example, Wuhan implemented a strict travel ban, and its 11 million residents were locked down on January 23, 2020, for more than 2 months. These measures have been effective in keeping rates of infection and mortality at a low level. As of 30 August 2020, mainland China had reported 85,048 confirmed cases and 4,634 deaths.^1^

However, these stringent pandemic-control rules have had a major adverse impact on the economy and individuals' daily lives. In the first half of 2020, the gross domestic product (GDP) of China shrank by 1.6%,^2^ which was the first contraction since the end of the Cultural Revolution in 1976. In addition, many people in China have experienced employment problems, such as (threats of) job loss, a reduction in their incomes, and/or working hours, and less regular work patterns. In this context, despite the incidence of COVID-19 being relatively low in China, it is possible that the shock to the economy and people's livelihoods may have created a general state of COVID-19 worry in many people.

Worry is defined as a series of uncontrolled thoughts that elicit negative feelings and a high level of anxiety and distress, which are linked to the fear of uncertain and probably undesirable outcomes (1). Several studies have found that COVID-19 worry has been a key cause of psychological distress during the pandemic, and a higher level of worry has been linked to a higher likelihood of mental health problems (2–6). According to a nationwide survey in the United States in late March 2020, ~45% of respondents reported that COVID-19 worry had a negative effect on their mental health (7). Similarly, in the United Kingdom, COVID-19 worry was found to be significantly associated with psychological distress (6). An online study on Russian-speaking healthcare workers also found that the increase in anxiety concerns about COVID-19 was associated with an increase in psychological distress (8). Moreover, it has been determined that even in regions with low rates of SARS-CoV-2 infection, COVID-19 worry significantly contributes to a general population's worsened mental health (9–11). As an illustration, a recent study in Hong Kong showed that compared to individuals who were less worried about COVID-19, those who were more worried about COVID-19 were more likely to have mental health problems (10). Similarly, Chan et al. (11) found that worries about being infected with SARS-CoV-2, and about the economy, their livelihoods, and their families' financial situation, had strong negative effects on the mental health of individuals in Hong Kong during the pandemic (11).

Furthermore, previous studies have reported that the effect of COVID-19 worry on mental health might vary between social groups with different demographic characteristics and economic statuses. In particular, sex, age, and socioeconomic status have been determined to be key factors mediating the impact of COVID-19 worry on mental health (2, 9, 12, 13). First, some studies have shown that women are more likely than men to suffer mental health problems due to COVID-19 worry (2, 14). For instance, a recent study in Norway found that COVID-19 worry can partially explain why women reported experiencing more psychological distress than men during the pandemic (2). A similar finding emerged from a survey of university students in Germany: that the mental well-being of female students was more adversely affected than that of male students' by concerns about the future in the pandemic (14). Second, there is evidence that the effect of COVID-19 worry on mental health is more significant among younger people than older people (13, 15–17). For example, based on a nationwide survey in the United States, Wilson et al. (13) found that COVID-19 worry was significantly positively associated with psychological distress in younger adults (aged 18–49), but not significantly associated with psychological distress in older adults (aged 50 and older) (13). Additionally, a study in Canada found that compared to older people (aged over 35), younger people (aged 15–34) reported more COVID-19 worries and had more maladaptive health habits, and therefore had a lower level of mental well-being (17). Third, the mental health of individuals with low socioeconomic status is more likely to be negatively affected by COVID-19 worry than that of those with higher socioeconomic status (6, 9). This is explained by the fact that compared to socioeconomically advantaged individuals, socioeconomically disadvantaged individuals (such as unemployed people) have less access to resources (such as financial resources and social support) (2), and therefore are more vulnerable to negative effects of the pandemic on the economy, their daily lives, and their physical/mental health (9).

Although many studies have analyzed the correlations between COVID-19 worry and mental health, most have focused on the general population and healthcare workers (e.g., 2–6) rather than on the economically active population (EAP). The study of Chan et al. (11) was an exception, as their online survey in Hong Kong revealed that compared to the economically inactive population, the mental health of the EAP was more likely to be negatively affected by COVID-19 worry (11). However, they did not further examine the heterogeneity of the effect, and thus did not examine whether COVID-19 worry has varying effects on the mental health of different groups within the EAP.

Based on the above findings, we posit that although China has effectively controlled the spread of SARS-CoV-2, COVID-19 worry may nevertheless indirectly affect the mental health of the EAP. Moreover, the pandemic has diverse effects on individuals' employment, and income is divergent. For example, although the COVID-19 pandemic and the government's response to it have harmed China's economy in general, they have boosted platform economy development by increasing demands for online services (18). Therefore, while some groups within the EAP have faced the problems of job loss, or reductions in working hours and income, platform workers have generally experienced increased employment opportunities and incomes. There is therefore an urgent need to explore the varied impacts of COVID-19 worry on the mental health of various groups within the EAP.

This study examines the correlations between COVID-19 worry and the mental health of the EAP in Guangdong province, China. The EAP comprises employed, self-employed, and unemployed people who are seeking employment, and not the economically inactive population, such as students, retired persons, and homemakers. Specifically, this study aims to determine the validity of the following hypotheses.

H1. COVID-19 worry has an adverse effect on the mental health of the EAP.
H2. Sex mediates the effect of COVID-19 worry on the mental health of the EAP: COVID-19 worry has a more adverse effect on the mental health of women than on that of men.
H3. Age mediates the effect of COVID-19 worry on the mental health of the EAP: COVID-19 worry has a more adverse effect on the mental health of younger age groups
H4. Economic activity status mediates the effect of COVID-19 worry on the mental health of the EAP: COVID-19 worry has a more adverse effect on the mental health of unemployed individuals than on that of (self-)employed individuals.
H5. Occupation type mediates the effect of COVID-19 worry on the mental health of the EAP: COVID-19 worry has a less adverse effect on the mental health of platform workers than on that of other employees.

## Methods

### Participants and Procedure

A cross-sectional study was conducted among the EAP in Guangdong province from 1 August to 30 September 30, 2020. Guangdong is the most flourishing and wealthy province in China and had a population of ~126 million in 2020.^3^ The first confirmed case of COVID-19 in Guangdong province was announced on 19 January 2020. As of 23 August 2020, Guangdong province had reported a total of 1,727 confirmed cases of COVID-19.^4^ Although rates of SARS-CoV-2 infection have been relatively low compared to some Western countries, the strict pandemic control rules have significantly affected the economy. In the first half of 2020, the GDP of Guangdong fell by 2.5% year-on-year.^5^

The eligibility criteria were (1) currently living in Guangdong, (2) aged 16 or older, and (3) employed, self-employed, or unemployed but seeking employment. An anonymous online questionnaire was distributed, and ~1,708 were completed by participants. After deleting incomplete responses, we obtained a total of 1,584 valid questionnaires (a response rate of 92.74%).

The respondents comprised 851 (53.72%) men and 733 (46.28%) women. In total 1,067 (67.36%) were aged 16–35, 267 (16.86%) were aged 36–45, and 250 (15.78%) were aged 46–70. Most of the respondents (59.09%) held rural *hukou* (household registration),^6^ and 53.03% were unmarried. The majority of participants (60.41%) reported that they were healthy. Moreover, 967 (61.04%) were employees (other than platform workers), 193 (12.18%) were platform workers, 254 (16.04%) were self-employed, and 170 (10.73%) were unemployed. The average per capital monthly family income was 2,536.68 RMB (~402.54 USD). The average income of the respondents constituted 43.92% of their total household income.

### Measurements

#### Worry About COVID-19

Because of the repeated COVID-19 outbreaks at a time when no vaccines or effective medicines were available, there was a widespread worry that the effects of the COVID-19 pandemic would be long-lasting and uncontrollable (19). Given this context, COVID-19 worry was measured by asking “How worried are you that the COVID-19 pandemic cannot be contained?” Responses ranged from 1 (*not at all worried*) to 5 (*extremely worried*).

#### Mental Health

Participants' mental health, the focal-dependent variable of this study, was measured using the Chinese 12-item General Health Questionnaire (GHQ-12), which is commonly used to screen for mental health problems (20–22). The questionnaire comprised of 12 items that assess respondents' anxiety and depression symptoms (e.g., “I have lost much sleep due to worry” and “I always feel stressed”), social dysfunction (e.g., “I am able to concentrate when doing things” [reverse coded]), and loss of confidence (e.g., “I have been thinking I am a worthless person”) (23). Each item was graded on a 4-point Likert scale ranging from 1 (*not at all*) to 4 (*much more/less than usual*). We used the “0-0-1-1” scoring method to sum the item scores (24) into a score scale ranging from 0 to 12. A higher score on the GHQ-12 indicates poorer mental health (24).

#### Sociodemographic Factors

We collected the participants' basic sociodemographic information, such as age, sex, marital status, number of children, years of education, *hukou* status, per capita monthly household income, and the proportion of their income in total household income. We also recorded the participants' current economic activity status, such as employees (other than a platform worker), platform worker, self-employed, and unemployed but seeking employment.

#### Lifestyle Factors

We collected information on health-related aspects of the participants' lifestyles, namely the frequencies of drinking alcohol and smoking. In addition, we recorded participants' perceived health status and whether or not the participant was living alone (Yes = 0, No = 1).

### Statistical Analyses

First, descriptive statistics were generated for the sample. Second, we ran ordinary least squares (OLS) regression models to account for covariates. Third, we examined the heterogeneity of the effect of COVID-19 worry on the mental health of our sample of the EAP.

## Model Specification

The empirical model presented here examines the correlations between COVID-19 worry and the mental health of our EAP sample. In the model, the GHQ-12 mental health score is the explained variable, and COVID-19 worry is the key explanatory variable. Sociodemographic and lifestyle characteristics are included as control variables. The equation is as follows:


$$\begin{array}{l} {mental\;health}_{i}=\alpha +\beta {worry}_{i}+\gamma V_{id}+\delta V_{il\;}+\varepsilon_{i} \end{array}$$

where the subscript ***i*** refers to individuals within the EAP, **V**_**id**_ are sociodemographic variables, **V**_**il**_ are lifestyle variables, and **ε** is a random error term. The summary statistics for the variables are presented in Table 1.

**Table 1:** Statistics of the variables.

**Variables**	**Obs**.	**Mean**	**Std. Dev**.	**Min**	**Max**
Mental health	1,584	1.441	1.912	0	10
COVID-19 worry	1,584	3.262	1.170	1	5
Sex	1,584	0.463	0.499	0	1
Age	1,584	32.203	10.622	16	70
Hukou	1,584	0.409	0.492	0	1
Years of education	1,584	12.310	3.130	0	19
Number of children	1,584	0.833	1.056	0	5
Per capita monthly household income	1,584	2,536.676	2,196.731	83.333	8,333.333
Proportion of respondent's income in total household income	1,584	43.917	25.360	0	100
Economic activity status	1,584	1.726	1.043	1	4
Drinking alcohol	1,584	1.259	0.499	1	3
Smoking	1,584	1.525	0.965	1	4
Living alone	1,584	0.770	0.421	0	1
Perceived health	1,584	1.466	0.624	1	3

## Results

### Descriptive Data on GHQ-12 Score and COVID-19 Worry of the Participants

Table 2 reports the means and standard deviations (SDs) of the GHQ-12 mental health scores for each sociodemographic category. In the total sample, the average GHQ-12 score was 1.44. Regarding COVID-19 worry, only 6.75% of participants reported that they were “not at all worried” about the containment of the pandemic; 20.39% reported they were “not very worried,” 30.81% reported they were “moderately worried,” 23.99% reported they were “very worried,” and 18.06% reported they were “extremely worried.” Therefore, COVID-19 worry was widespread among our sample of the EAP in Guangdong province. Furthermore, the descriptive statistics show that participants who were “very worried” or “extremely worried' had higher GHQ-12 mental health scores (see Table 2).

**Table 2:** The GHQ-12 mental health scores for each sociodemographic category.

	**Sample size**	**Mean**	**Std. Dev**.
**Total**	1,584	1.441	1.911
COVID-19 Worry			
Not worried at all	107	1.327	2.086
Not very worried	323	1.108	1.667
Moderately worried	488	1.264	1.820
Very worried	380	1.444	1.834
Extremely worried	286	2.157	2.166
Sex			
Male	851	1.461	1.886
Female	733	1.417	1.941
Age			
16–35	1,067	1.644	2.021
36–45	267	1.262	1.740
46–70	250	0.764	1.351
Hukou			
Rural hukou	936	1.362	1.841
Urban hukou	648	1.555	2.004
Educational level			
Junior high school or below	423	1.184	1.705
Senior high school	543	1.383	1.844
College/university degrees or above	618	1.668	2.072
Number of children			
Unmarried	840	1.677	2.044
Zero or one	268	1.485	1.914
Two or more	476	1.000	1.561
Frequency of drinking			
Less than once a month	1,219	1.408	1.880
Once a month to twice a day	320	1.462	1.918
More than twice a day	45	2.177	2.507
Smoking			
Do not smoke.	1,190	1.447	1.913
Started smoking in the past year.	60	1.800	1.981
Have smoked for more than 1 year.	231	1.259	1.794
Had quit smoking.	103	1.572	2.084
Perceived health status			
Very healthy	957	1.289	1.850
Moderately healthy	516	1.529	1.886
Not healthy	111	2.342	2.258
Economic activity status			
Employees (other than platform workers)	967	1.462	1.955
Platform workers	193	1.450	1.811
Self-employed	254	1.102	1.541
Unemployed but seeking employment	170	1.817	2.185

### Correlation Between COVID-19 Worry and Mental Health of the Participants

Table 3 presents the factors influencing the participants' mental health. Model 1 includes the variables of COVID-19 worry and mental health. Models 2 and 3 also include sociodemographic or lifestyle variables, respectively. In Model 1, a significant association between COVID-19 worry and GHQ-12 mental health score was observed. Moreover, the coefficients and significance levels of the COVID-19 worry variable were similar in Models 2 and 3. Specifically, the coefficients of COVID-19 worry were 0.251, 0.224, and 0.221 in Models 1, 2, and 3, respectively (all *p* < 0.01). Therefore, the regression results support H1, that is, COVID-19 worry has an adverse effect on the mental health of the EAP.

**Table 3:** Effect of COVID-19 worry on the mental health of EAP.

**Variables**	**Model 1**		**Model 2**		**Model 3**	
	**β (SE)**	**t**	**β (SE)**	**t**	**β (SE)**	**t**
COVID-19 worry	0.251^**^ (0.041)	6.173	0.224^**^ (0.041)	5.499	0.221^**^ (0.040)	5.457
Female (Male = 0)			0.008 (0.100)	0.079	−0.054 (0.111)	−0.476
Age			−0.020^**^ (0.007)	−2.908	−0.020^**^ (0.007)	−2.911
Urban Hukou (Rural Hukou = 0)			0.119 (0.097)	1.221	0.151 (0.097)	1.556
Years of education			0.002 (0.019)	0.125	0.003 (0.019)	0.186
Number of children			−0.173^*^ (0.068)	−2.565	−0.144^*^ (0.068)	−2.130
In(per capita monthly household income)			−0.071 (0.056)	−1.273	−0.123 (0.064)	−1.926
Percentage of the respondent's income in total household income			0.008^**^ (0.002)	4.072	0.008^**^ (0.002)	3.925
Self-employed			−0.259 (0.134)	−1.929	−0.280^*^ (0.134)	−2.092
Platform workers			0.059 (0.151)	0.392	0.065 (0.149)	0.432
Unemployed			0.401^*^ (0.159)	2.521	0.343^*^ (0.158)	1.807
Drinking alcohol (less than once a month = 0)						
Once a month to twice a day					0.137 (0.126)	1.086
More than twice a day					0.649^*^ (0.285)	2.277
Smoking (do not smoke=0)						
Started smoking in the past year					0.109 (0.254)	0.431
Have smoked for more than 1 year					−0.039 (0.158)	−0.247
Had quit smoking					0.216 (0.200)	1.081
Not living alone (Living alone = 0)					−0.153 (0.135)	−1.139
Perceived health (Healthy=0)						
Moderately healthy					0.165 (0.102)	1.623
Not healthy					0.981^**^ (0.186)	5.285
Constant	0.624^**^ (0.141)	4.434	1.564^**^ (0.552)	2.835	1.902^**^ (0.631)	3.014
Observations	1,584		1,584		1,584	
R-squared	0.024		0.068		0.091	

*Significance level,*

** *p < 0.01,*

* *p < 0.05*.

### Other Factors Associated With the Participants' Mental Health

As shown in Table 3, age, family economic burden (the percentage of total family income that is an individual's income), current economic activity status, number of children, frequency of drinking alcohol, and self-perceived health status were strongly correlated with mental health. First, the coefficient of age was −0.020 (*p* < 0.01), indicating that age had a significant positive association with mental health. Second, as expected, the family economic burden had a negative relationship with mental health (*p* < 0.001). That is to say, if a family economy is highly dependent on the income of the respondent, the respondent is more likely to have mental health problems during the pandemic. Third, the mental health of participants who were employees was significantly worse than that of participants who were self-employed (*p* < 0.05) but better than that of participants who were unemployed persons (*p* < 0.05). Fourth, having more children was positively correlated with mental health. Fifth, those who consumed alcohol at least twice a day were more likely to have psychological distress than those who consumed alcohol less than once a month (*p* < 0.05). Finally, compared to participants who perceived themselves as healthy, those who perceived themselves as not healthy were more likely to have poorer mental health (*p* < 0.01).

### Group Comparison

To examine the heterogeneity of the effect of COVID-19 worry on the mental health of the EAP, we divided the entire sample into subgroups by sex, age, and economic activity status. Table 4 reports the regression results by sex. COVID-19 worry was strongly correlated with both men's and women's mental health (both *p* < 0.01). Contrary to our expectations, however, we found the coefficient of the COVID-19 worry variable was larger for male (0.264) than for female (0.173) participants, indicating that COVID-19 worry had a more adverse effect on the mental health of men than on that of women. Therefore, H2 was rejected.

**Table 4:** Effect of COVID-19 worry on mental health, by gender.

**Variables**	**Men**		**Women**	
	**β (SE)**	**t**	**β (SE)**	**t**
COVID-19 worry	0.264^**^ (0.056)	4.728	0.173^**^ (0.059)	2.925
Constant	1.261 (0.857)	1.472	2.393^*^ (0.960)	2.493
Observations	851		733	
R-squared	0.118		0.097	

*Significance level,*

** *p < 0.01,*

* *p < 0.05*.

Table 5 presents regression results of the subgroups of participants who were aged 16–35, 36–45, and 46–70. COVID-19 worry was strongly correlated with mental health in the 16–35-year-old group (*p* < 0.01) and the 36–45-year-old group (*p* < 0.05) but was not correlated with mental health in the 46–70-year-old group (*p* > 0.1). This result supports H3. Consistent with previous studies, we found that COVID-19 worry had a significantly adverse effect on the mental health of the younger age-groups but not on that of the older group.

**Table 5:** Effect of COVID-19 worry on mental health, by age.

**Variables**	**16–35**		**36–45**		**46–70**	
	**β (SE)**	**t**	**β (SE)**	**t**	**β (SE)**	**t**
COVID-19 worry	0.280^**^ (0.053)	5.319	0.183^*^ (0.091)	2.016	0.041 (0.079)	0.516
Constant	0.413 (0.788)	0.524	4.494^**^ (1.514)	2.968	1.241 (1.110)	1.118
Observations	1,067		267		250	
R-squared	0.081		0.126		0.130	

*Significance level,*

** *p < 0.01,*

* *p < 0.05*.

Table 6 shows the regression results of the subsamples of participants who were employees (other than platform workers), platform workers, self-employed, or unemployed. COVID-19 worry was strongly correlated with the mental health of participants who were employees (*p* < 0.01) or unemployed (*p* < 0.05), with coefficients of 0.277 and 0.324, respectively. This implies that COVID-19 worry had a more adverse effect on the mental health of participants who were unemployed than on that of those who were employees. Hence, the results support H4. We also found that COVID-19 worry was not correlated with the mental health of participants who were platform workers, which supports H5.

**Table 6:** Effect of COVID-19 worry on mental health, by employment status.

**Variables**	**Employees**		**Platform workers**		**Self-employed**		**Unemployed**	
	**β (SE)**	**t**	**β (SE)**	**t**	**β (SE)**	**t**	**β (SE)**	**t**
COVID-19 worry	0.277^**^ (0.054)	5.105	0.088 (0.110)	0.799	0.149 (0.081)	1.851	0.324^*^ (0.137)	2.357
Constant	0.904 (0.822)	1.101	1.545 (1.585)	0.975	1.953 (1.214)	1.609	2.400 (2.125)	1.129
Observations	967		193		254		170	
R-squared	0.083		0.105		0.121		0.230	

*Significance level,*

** *p < 0.01,*

* *p < 0.05*.

## Discussion

This study examined how COVID-19 worry affected the mental health of a sample of the EAP in Guangdong province, China. We found that there was widespread worry about the containment of COVID-19 during the pandemic. Approximately 42.05% of the participants reported being “very worried” or “extremely worried” about the pandemic. The overall level of COVID-19 worry in the present study was similar to some previous findings in Greater China. For example, an online survey in Taiwan found that 51.7% of respondents reported high levels of worry about COVID-19 in April 2020 (25). Additionally, a survey in Hong Kong from late April to early May 2020 showed that 57.6% of respondents reported they are worried about the COVID-19 pandemic (9). During the early stage of the pandemic, effective vaccines were absent, and new outbreaks may occur at any time, which contributed to the high level of worry related to COVID-19. Moreover, as predicted, we found that compared to participants who were less worried about the containment of the pandemic, those who were more worried about the containment of the pandemic had higher GHQ-12 mental health scores, suggesting that COVID-19 worry was negatively correlated with mental health. This finding reveals that even in countries with very low infection rates, the COVID-19 pandemic may still negatively affect the mental health of EAPs *via* worry. Echoing prior studies (6, 26), this study shows that COVID-19 worry placed an additional psychological burden on the EAP.

This study further reveals that the correlation between COVID-19 worry and mental health was heterogeneous across different demographic groups. For example, although COVID-19 worry had a significantly adverse effect on the mental health of both men and women, this adverse effect was greater in men. This finding is not consistent with previous studies of sex differences in Norway and Germany, which have found that women were more affected by COVID-19 worry (2, 14). This inconsistency may be attributable to the more traditional roles of the sexes in China, where there has been a resurgence of the Confucian patriarchal tradition (27). In Chinese society, men are supposed to shoulder most of the responsibility to support their families and secure family-wage employment (28). Thus, in the context of economic uncertainty, worrying that the COVID-19 pandemic would not be controlled might have created more of a psychological burden for men than for women. In addition, this study shows that COVID-19 worry was significantly associated with worse mental health in younger people (aged 16–45) but was not significantly associated with worse mental health in older people (aged 46–70). This finding echoes most of the literature data, which has shown that compared to younger people, older people are more experienced in regulating their emotions (29) and using more effective adaptive strategies (16), and are thus less likely to be negatively affected by worry about the consequences of COVID-19 (13, 17, 30).

By comparing groups with different economic activity statuses, this study also reveals that there was heterogeneity in the effect of COVID-19 worry on the mental health of the EAP. As hypothesized, COVID-19 worry was more strongly associated with worse mental health in participants who were unemployed than in those who were employed or self-employed. This is consistent with the fact that compared to employed people, unemployed people generally face more financial hardship and livelihood insecurity and are thus more vulnerable to the negative consequences of COVID-19 worry. This result is in line with previous findings in South Africa, Bangladeshi, and the United States, which showed that adults who kept their paid employment during the COVID-19 pandemic are less likely to have mental health problems than those who lost their jobs (31–33). In addition, although the COVID-19 worry was negatively correlated with the mental health of self-employed persons, the correlation was not statistically significant (*p* > 0.05). This finding may be attributable to the fact that self-employed people in China usually own certain means of production, such as small shops or a piece of farmland, which gives them a relatively higher capacity than employees to sustain their livelihoods under economic uncertainty. Interestingly, we found that COVID-19 worry did not have a significant effect on the mental health of platform workers. This finding is reasonable because with the burgeoning of the platform economy during the pandemic, the employment and incomes of platform workers were generally stable or improved, which may have buffered the negative effect of COVID-19 worry on their mental health. Therefore, we argue that COVID-19 worry created new inequalities in mental health among the EAP.

## Conclusion

Based on an online survey in Guangdong province, this study found that despite the low rates of SARS-CoV-2 infection in mainland China, there was widespread worry among the EAP about the containment of the COVID-19 pandemic, and that this worry negatively influenced their mental health. The adverse effect of COVID-19 worry on mental health was greater among men, younger individuals (aged 18–45), and unemployed persons. One key policy implication of the present study is that in addition to material support, governments should provide more public reassurance and psychological support to the EAP (especially younger people, men, and unemployed persons) to help them cope with COVID-19 worry, as this will diminish the negative impacts of the pandemic on their mental well-being.

## Data Availability Statement

The raw data supporting the conclusions of this article will be made available by the authors, without undue reservation.

## Ethics Statement

The studies involving human participants were reviewed and approved by the Research Ethics Committee of the South China Normal University. Written informed consent for participation was not required for this study in accordance with the national legislation and the institutional requirements.

## Author Contributions

XY conceptualized the study, interpreted the data, and wrote the initial draft of the manuscript. LZ contributed to the study design, data analysis and interpretation, and the drafting of the manuscript. Both authors have critically revised and approved the final draft of the manuscript.

## Funding

This study was supported by the Outstanding Youth Project for Scientific Research from the Hunan Provincial Department of Education (Project No.: 20B552).

## Conflict of Interest

The authors declare that the research was conducted in the absence of any commercial or financial relationships that could be construed as a potential conflict of interest.

## Publisher's Note

All claims expressed in this article are solely those of the authors and do not necessarily represent those of their affiliated organizations, or those of the publisher, the editors and the reviewers. Any product that may be evaluated in this article, or claim that may be made by its manufacturer, is not guaranteed or endorsed by the publisher.

## Footnotes

^1^China National Health Commission. *The latest situation of COVID-19 as of 24:00 on August 30* [in Chinese]. August 31, 2020, available at http://www.gov.cn/xinwen/2020-08/31/content_5538643.htm

^2^National Bureau of Statistics of China. *The semi-annual report on China's economy in 2020* [in Chinese]. July 16, 2020, available at http://www.gov.cn/zhuanti/2020zgjjbnb/index.htm

^3^Nanfang Daily. *The resident population of Guangdong reached 126 million, with an increase of 21.71 million in 10 years* [in Chinese]. May 12, 2021, available at http://www.gd.gov.cn/gdywdt/bmdt/content/post_3279829.html

^4^Guangdong Provincial Health Commission. *COVID-19 situation in Guangdong province on August 24, 2020* [in Chinese]. August 24, 2020, available at: http://www.gd.gov.cn/gdywdt/zwzt/yqfk/qktb/content/post_3070994.html

^5^Guangdong Provincial Bureau of Statistics. *The economy of Guangdong in the first half of 2020* [in Chinese]. July 20, 2020, available at: http://www.gd.gov.cn/zwgk/sjfb/sjkx/content/post_3047584.html

^6^*Hukou* is a household registration system used in mainland China. It records a person's residency as agricultural (rural *hukou*) or non-agricultural (urban *hukou*). The *hukou* system has been a major source of social inequality; for example, employees holding urban *hukou* have generally received more labor protection and social welfare (such as pensions and health care) than their counterparts holding rural *hukou*. In this study, the *hukou* variable was coded as a dummy (rural = 0, urban = 1). As shown in Table 1, the mean *hukou* was 0.409, which means that 40.9% of respondents held urban *hukou* and 59.1% held rural *hukou*.
